# The segmentation clock mechanism moves up a notch

**DOI:** 10.1016/j.tcb.2010.07.001

**Published:** 2010-10

**Authors:** Sarah Gibb, Miguel Maroto, J. Kim Dale

**Affiliations:** College of Life Sciences, University of Dundee, Dundee, DD1 5EH, Scotland, UK

## Abstract

The vertebrate segmentation clock is a molecular oscillator that regulates the periodicity of somite formation. Three signalling pathways have been proposed to underlie the molecular mechanism of the oscillator, namely the Notch, Wnt and Fgf pathways. Characterizing the roles and hierarchy of these three pathways in the oscillator mechanism is currently the focus of intense research. Recent publications report the first identification of a molecular mechanism involved in the regulation of the pace of this oscillator. We review these and other recent findings regarding the interaction between the three pathways in the oscillator mechanism that have significantly expanded our understanding of the segmentation clock.

## Somitogenesis

A segmented body plan is a characteristic feature of all vertebrate and many invertebrate species. The process of segmentation is initiated very early in the developing vertebrate embryo and involves the generation of repeated segments, or somites, along the anterior to posterior axis. Somites play a key role in subsequent body patterning by governing the formation of all adult segmented structures. Disruption of the segmentation process in vertebrates can result in conditions characterised by fusion of the ribs and spinal deformities or truncations [1]. Occurrence of syndromes and disorders that include abnormal vertebral segmentation, such as Spondylocostal Dysostoses (a group of severe axial skeletal malformation diseases likely due to defects in signalling during embryonic development), is quite common during human development, although their prevalence remains difficult to ascertain [1,2]. It is clear that the study of somite formation in model animals presents the best way to investigate this process. We can then make inferences from those findings with regard to the molecular basis of human segmentation.

**Figure 1:**
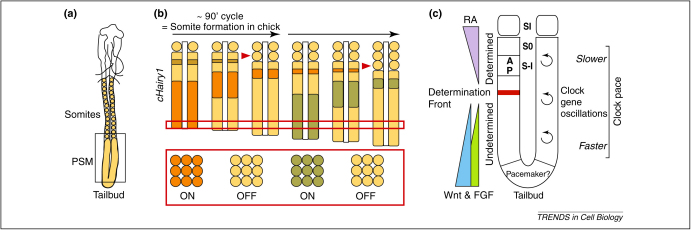
**The vertebrate segmentation clock oscillator.** **(a)** Schematic showing the dorsal view of a 2 day-old chicken embryo, and the position of somites and the PSM that flank the axial neural tube. As somites bud off the anterior end of the PSM, new cells are recruited into the posterior PSM from the progenitor cells in the tail bud [4–6]. **(b)** The PSM tissue in a is magnified in b to illustrate the evidence for an oscillator underlying vertebrate segmentation. Periodic waves of transcriptional expression of the *cHairy1* gene (successive waves shown in different colours) across the PSM share the same periodicity as somite formation, 90 minutes in chick [19]. The red box is magnified at the bottom of this figure to illustrate what this process means at the level of individual PSM cells. During each oscillation, individual cells within the PSM turn on and off the gene. This dynamic expression at the level of single cells, by virtue of being synchronised across the PSM, results in apparent ‘waves’ of gene expression that ‘move’ across the PSM (top part of panel). The cells themselves suffer very little anterior movement at all. However, as somites bud off the rostral PSM and new cells enter the caudal PSM, individual cells within the PSM become progressively more anteriorly displaced in the PSM (see the red box in the top part of the panel). **(c)** A schematic diagram integrating the domains of various signalling activities in the PSM – the wavefront of determination on the left hand side, and the clock on the right. The system of opposing gradients of Fgf (green), Wnt (blue) and retinoic acid (RA - purple) signalling in the PSM positions the determination front (red) along the PSM [18]. The determination front marks the position where the next prospective boundary will form, thereby defining somite size [18]. As these cells mature, the anterior (A) and posterior (P) somite compartments become specified. In the most rostral PSM the definitive morphological boundary of the next prospective somite forms. As indicated on the right side of the diagram, within this same PSM tissue, waves of Notch, Fgf, and Wnt cyclic gene expression controlled by the segmentation clock oscillator traverse the PSM periodically (black spiral symbol). The oscillations slow down as they reach the rostral PSM. Wnt activity appears to act as (part of) the pacemaker mechanism to regulate the periodicity of cyclic gene oscillations [59]. Prospective somites in the PSM are numbered with somite S0 being the forming somite and the somites next to form labelled in negative Roman numerals, S-I *etc*[90]. Segmented somites are numbered in positive Roman numerals, with SI being the most recently formed somite.

Somites are progressively pinched off in pairs from the anterior end of two rods of mesenchymal tissue called presomitic mesoderm (PSM), which lie either side of the caudal neural tube at the posterior end of the embryo [3] (Figure 1A). The PSM tissue is replenished by continuous recruitment of cells from a region located at the posterior end of the embryo called the tail bud, which contains an embryonic stem cell population [4–6] (Figure 1A). Thus, somite formation occurs in concert with extension of the body axis at the posterior end. This process continues with precision until the final number of somites is reached. Both the total number of somites formed and the periodicity with which they are produced are species-specific parameters [3,7,8].

The somitogenesis process is an exquisitely organised, multistep process. Newly recruited mesenchymal cells enter the posterior PSM, and gradually mature, becoming progressively displaced anteriorly in the PSM, as somites are pinched off the anterior end of this tissue (Figure 1B). Midway along the PSM, at the so-called determination front (see below), cells become grouped together, and are thereby allocated to prospective somitic units in a periodic fashion (Figure 1C). The rostral and caudal halves of each prospective somite are specified in the most mature anterior half of the PSM (Figure 1C). Formation of the morphological somite boundary occurs in the anterior limit of the PSM (Figure 1C). Finally, after their formation, somites differentiate into a number of tissues, namely the vertebrae, ribs, tendons, intercostal and skeletal muscles as well as the dermis of the back. The mechanisms underlying many of these aspects of somite formation have been reviewed extensively elsewhere [9–15]. In this review, we will discuss our current understanding of the molecular mechanism underlying the generation and tight temporal control of periodicity in the PSM, which is believed to be regulated by a molecular oscillator termed the segmentation clock. We will also discuss what is known about the pacemaker that regulates the speed of the clock oscillations.

## The segmentation clock

Due to the requirement for a specific number of somites to form in a given time period it is critical that somite formation is under tight temporal control. Theoretical models postulated to explain the periodic production of somites include the Clock and Wavefront model, which proposed the existence of an oscillator and a wavefront of maturation operating in the PSM [16] (reviewed in [17]). Briefly, in this model the wavefront represents the anterior to posterior progression of development of the embryo. Thus, this wavefront of maturation sweeps along the body axis in concert with extension of the trunk and tail and in particular it governs the maturation of the PSM to become somites. The activity of the wavefront is gated by an oscillator, or clock, acting in the PSM cells: a somite unit forms only when the wavefront of maturation reaches a group of cells in the appropriate phase of the clock. In this model the size of each somite is determined by the speed of the wavefront whereas the rate of somite formation is controlled by the frequency of the oscillator.

The molecular evidence supporting the existence of a wavefront of maturation in the PSM of a variety of vertebrate species relies on the intersecting gradients and cross-regulatory activities of three signal transduction pathways, namely Fgf, Wnt and Retinoic acid (RA) (Figure 1C). The point of intersection of these gradients, the so-called determination front, marks the position where the next prospective somite boundary will form, thereby regulating somite size (Figure 1C). This subject has been reviewed elsewhere, and will not be covered in this review [18]. Molecular evidence supporting the existence of an oscillator in the PSM similar to that proposed in the model came with the discovery of the first of the cyclic genes, *c-Hairy1* [19]. This gene was shown to display dynamic waves of mRNA expression that sweep caudorostrally across the length of the chick (*Gallus gallus*) PSM (Figure 1). These waves of expression are dynamic and cyclical, with the same periodicity as that of somite formation [19].

**Figure 2:**
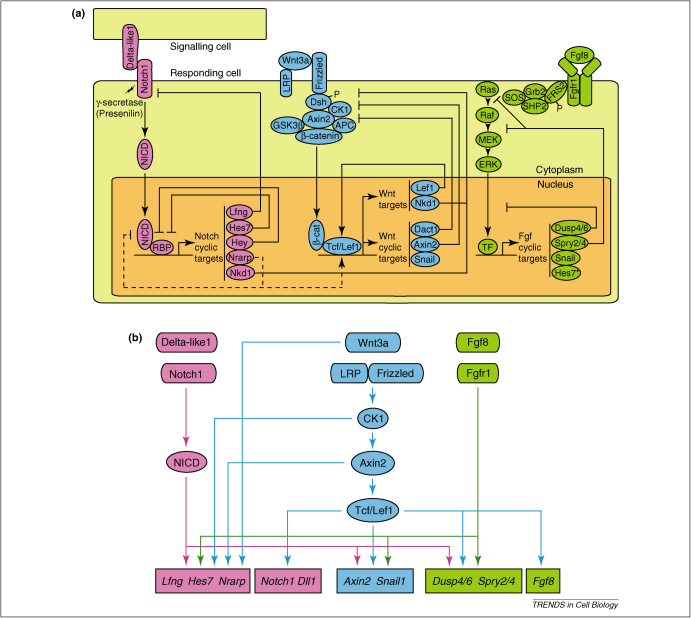
**Crosstalk between the three distinct oscillators that function in the mouse PSM.** **(a)** Schematic diagram of a signalling and a responding cell in the mouse PSM, detailing cyclic genes of the Notch, Fgf and Wnt pathways. Notch and Fgf-regulated cyclic genes oscillate asynchronously to cyclic genes of the Wnt pathway [56]. A large number of the cyclic genes are involved in negative feedback loops [3]. The very basic circuitry of the three signalling pathways is represented here. N.B. Hes7 expression is Fgf-dependent in the caudal PSM only. Hashed line represents interactions that to date have only been shown in tissues outside of the PSM [75,78]. **(b)** The crosstalk indicated between the three pathways in this part of the figure are interactions that have been demonstrated specifically within the PSM largely through the analysis of mouse mutants and some pharmacological studies [33,53,54,56,58–60,70,79]. As such, it is not clear if these interactions are direct. These analyses rely on analysis of mRNA expression of the various pathway components as indicated by italicised gene names. The interactions are colour coded such that regulation of the Wnt or Fgf pathway components by the Notch pathway are indicated in pink, input from the Wnt signalling pathway to the regulation of Notch or Fgf components are in blue. Regulation of the Notch or Wnt pathway components by the Fgf pathway is indicated in green. Dll1, Delta-like 1; DACT1, dapper homologue 1; DSH, dishevelled; DUSP4/6, dual specificity phosphatase 4/6; FGFR1, FGF receptor 1; GSK3, glycogen synthase kinase 3; Hes7, hairy and enhancer of split related 7; LFNG, lunatic fringe; LRP, low density lipoprotein receptor-related protein; NICD, Notch intracellular domain; NKD1, naked cuticle 1 homologue; Nrarp, Notch-regulated ankyrin repeat protein; RBP, recombination signal binding protein for immunoglobulin kappa J region; Spry 2/4, sprouty 2 and sprouty 4; CKI, Casein kinase I. ERK, mitogen-activated protein kinase 1 (MAPKK); MEK, mitogen-activated protein (MAPK); Raf, MAP kinase kinase kinase (MAP3K); Ras, small GTPase; GRB2, growth factor receptor-bound protein 2; SHP2, Src homology region 2-containing protein tyrosine phosphatase 2; SOS, son of sevenless; FRS2, Fibroblast growth factor receptor substrate 2; APC, protein encoded by the adenomatosis polyposis coli gene; Lef, Lymphoid enhancer-binding factor; Hey, Hairy/enhancer-of-split related with YRPW motif protein; TF, Transcription factor complex.

Since the initial description of the expression of *cHairy1*, multiple other cyclic genes have been shown to oscillate at the mRNA level in the PSM of chick, mouse (*Mus musculus*) and zebrafish (*Danio rerio*) embryos [3]. Among these cyclic genes are a group of Hairy/enhancer of split (Hes) genes which are downstream targets of the Notch pathway that encode transcriptional repressors. These repressors are likely to establish negative feedback loops by repressing their own transcription, as reviewed in [20] (Figure 2A). One of these factors, Hes7, has been shown to also oscillate at the protein level in the mouse PSM with a half-life of around 22 minutes [21]. Extending this half-life to 30 minutes, by introducing a lysine-to-arginine point mutation, resulted in a halt of Notch-based cyclic gene oscillations and disrupted somite formation [22], suggesting that the negative feedback loops generated by these Hes repressors are important for the generation and/or maintenance of the oscillations associated with somitogenesis. Negative feedback loops also appear to control cyclic gene oscillations within the chick and zebrafish PSM, where several Hes homologues also oscillate [3]. In zebrafish, *her1* and *her7* appear to be primarily responsible for regulating cyclic gene oscillations, as reviewed in [3,15], and this function is also reliant on short half-lives for transcripts and proteins of these two clock components [23]. Surprisingly, oscillations based on negative feedback loops generated by Hes genes are not confined to the PSM, but also occur in a variety of mouse cell lines with a periodicity that matches mouse somitogenesis [24]. This is consistent with the possibility that this oscillatory activity may well be a more universal phenomenon experienced by many cell and tissue types in the vertebrate body. Indeed, a recent study demonstrated that Hes1 also oscillates in mouse ES cells, and thereby biases the cell fate adopted by these stem cells [25].

In addition to the oscillation of Hes-related genes, a similar dynamic expression of other Notch pathway components has since been reported in the PSM of all vertebrate species studied [3,26–28]. Thus, for example, the Notch-modifying glycosyltransferase enzyme, Lunatic Fringe (*Lfng*), oscillates in the PSM of both mouse [29,30] (Figure 2A) and chick [30,31] in a Notch-dependent fashion [32–34]. Lfng glycosylates the Notch receptor, which modifies Notch–ligand interactions to inhibit or potentiate Notch signalling [35–38]. In the vertebrate embryo, the only Notch ligands expressed along the PSM belong to the Delta family. In addition to being a Notch target, misexpression studies have shown that in the vertebrate PSM, *Lfng* blocks Notch signalling and interferes with somitogenesis [32,39]. Acting in a negative feedback loop, *Lfng* thereby inhibits its own transcription, and that of the other cyclic genes [32] (Figure 2A). Since Lfng protein is unstable, this inhibition is transient, and hence, Lfng degradation allows for the next wave of transcription to pass along the PSM [32]. Thus, the implication of Notch in the mechanism of the molecular oscillator seems to be controlled by multiple levels of negative feedback by unstable downstream targets [3] (Figure 2A).

## The role of Notch in the oscillator and in segmentation

Since many of the cyclic genes are Notch targets, one possibility is that Notch lies at the heart of the vertebrate segmentation clock mechanism. Consistent with this idea, mice carrying mutations in any one of the genes encoding ligands, receptors or downstream effectors of the Notch pathway display severe segmentation defects [40]. However, even very severe Notch mutants still display an albeit disturbed and irregular segmented body plan, raising the possibility that segmentation can occur in the absence of Notch activity; RBPj is a crucial component of the Notch pathway and the *RBPj*-/- mouse still produces some asymmetrical condensations in the anterior PSM [41]. However, a recent report suggests that these condensations may result from residual RBPj-independent Notch activity present in the PSM [33]. Importantly, this same recent report has shown that in the complete absence of Notch activity, as occurs in the double *Psen1*-/-;*Psen2*-/- mutant line, or wild type mouse embryos cultured in the presence of gamma-secretase inhibitors, dynamic cyclic gene expression in the PSM is abolished, and somitogenesis is completely ablated, showing Notch is absolutely essential for both processes in this species [33] (and see Box 1).

Conversely, and in stark contrast to the situation described in the mouse, the primary role attributed to Notch during fish segmentation is to coordinate a Notch-independent oscillator (reviewed in [42,43]). This idea stems from data showing that the first few somites continue to form in zebrafish Notch pathway mutants, and that *her1/her7* expression is not lost in these zebrafish mutants, and, in addition, that pharmacological Notch inhibition does not abolish *her1/her7* expression, nor does it stop segmentation [44–46], as reviewed in [3,15,42,43]. In these circumstances, an alternative pathway may be acting in zebrafish as the main signalling cascade regulating dynamic cyclic gene expression. Indeed, in the zebrafish PSM, Fgf rather than Notch may be largely responsible for maintaining the oscillations by acting as the positive input for *her* expression and function, as suggested by the fact that the Fgf-regulated gene *her13.*2 regulates *her1/her7* oscillations [47]. If loss of Notch does not affect *her1*/*her*7 expression, how is it implicated in the synchrony of the oscillations? Aside from the *her* genes, the only other gene reported to cycle in the fish PSM is the Notch ligand, *deltaC* [48]. *her1/her7* negatively regulate *deltaC,* which could thereby lead to periodic Notch activation [49,50]. A simple oscillator model has been proposed that essentially relies on the coupling of *her* genes to periodic Notch activation, to maintain synchrony between PSM cells [23,48,51,52]. Hence, the Notch pathway would synchronise oscillations in the zebrafish PSM, while the negative feedback loops generated by *her1/her7* would represent the core of the fish segmentation clock oscillator, where Fgf activity would act as a key signal providing positive input to *her1/her7* expression [42].

## Wnt- and Fgf-regulated cyclic genes

In addition to the Notch pathway, components of the Wnt and Fgf signalling pathways also oscillate in the mouse PSM (Figure 2A) [3]. These include several negative regulators, such as *mAxin2* [53], *mNkd1* [54], *mDact1* [55], *mSpry2* [56]*, mSpry4* [57], *mDusp4* [58] and *mDusp6* [56]. As such, negative feedback loops similar to those seen in the control of Notch-regulated oscillations, may also operate within these pathways (Figure 2A). Whereas the Wnt cyclic gene oscillations occur out of synchrony with the Notch regulated oscillations, the Fgf-regulated genes cycle in synchrony with Notch components [56]. Interestingly, recent work demonstrated a non-dynamic expression profile across the PSM for chick homologues of the mouse Wnt cyclic genes [59], suggesting either a species difference or, alternatively, that other Wnt components, non-homologous to mouse Wnt cyclic genes, may be oscillating in the chick PSM. With regard to Fgf-related cyclic genes, to date only chick *Snail2* has been shown to display different patterns of expression in the chick PSM [60]. So far, no Wnt or Fgf signalling components have been shown to cycle across the PSM of any other vertebrate species.

In summary, a common aspect underlying oscillations of the Notch, Wnt and Fgf cyclic genes appears to be the turnover of negative regulators. It remains unclear, however, to what extent the regulatory signalling pathways driving cyclic gene expression, or the cyclic genes themselves, are conserved across vertebrate species. This is clearly an important area to focus on in the future, since it is of key relevance to the elucidation of the etiologies of human pathologies associated with defective segmentation.

## Oscillator pacemaker

While the Notch, Wnt and Fgf pathways have been identified as the pathways that are required for expression of the clock genes identified to date, these oscillations also have to be entrained with a pacemaker to ensure that they occur with the correct periodicity. The molecular mechanism regulating the periodicity of cyclic gene oscillations is a key feature of the oscillator that has remained entirely obscure until very recently. Oscillation pace slows down in the rostral PSM [19] (Figure 1C), where levels of nuclear β-catenin are reduced compared to the rest of the PSM [61]. It has been suggested that a down-regulation of Wnt signalling may be required for the final arrest of oscillations in the rostral PSM [61]. Strikingly, molecular evidence consistent with this idea came from recent data showing attenuated Wnt signalling results in slower oscillations of the cyclic genes in both mouse and chick, suggesting that Wnt activity could be implicated in the regulation of oscillator pace [59]*.* In addition, reduced Wnt signalling in the PSM of the developing embryo, as occurs during development of the last few somites in chick, coincides with both an extended oscillation period and a complementary increase in somite formation time for these last somites [59,62]. Intriguingly, despite the fact that Wnt regulates Fgf signalling in the PSM (see below), the role of Wnt in regulating clock pace is not mediated by Fgf signalling in either chick or mouse, at least in the short term [59]. It remains to be seen how Wnt extends the oscillation period. Interestingly, ectopic activation of the Wnt pathway has no effect on oscillation pace [59,61,63], which may be due to the fact this tissue is already Wnt-saturated. It is also possible that the oscillations are occurring at maximum pace under normal conditions due to limitations imposed by the speed of production and turnover of both RNA and protein in such a short time frame.

## Molecular interactions and hierarchy between Notch, Fgf and Wnt

A central question within systems biology is how does the combined function of many genes within a network lead to higher levels of organization? A critical aspect of the segmentation clock oscillator is the need to understand the degree of crosstalk and hierarchy of each of the signalling pathways involved in clock regulation. This deeper level of understanding is of particular importance, given that each of the three pathways play multiple roles in the PSM. For example, Wnt signaling regulates cyclic gene expression (at least in mouse), the wavefront of determination, as well as oscillation pace (Figure 1C). While numerous interactions in non-developmental contexts have been reported between the Fgf, Notch and Wnt pathways at various levels of the signal transduction pathways [64–69], investigations of the interplay between these three pathways in the PSM have been largely confined to analyses of mutant mice, as reviewed in [43] (Figure 2B).

### Wnt and Notch

Data suggesting that Wnt signalling may occur upstream of Notch signalling in terms of the segmentation clock, include analyses of the *Wnt3a* hypomorphic mouse *vestigial tail* (*vt*), which revealed that cyclic expression of the Notch cyclic genes *mLfng* and *mNrarp* are Wnt3a-dependent [53,70]. In addition, misexpression of the Wnt-regulated cyclic gene *mAxin2* affects segmentation by ectopically activating *mLfng* transcription [53]. Furthermore, *Notch1* expression is lost in *Lef1*-/-;*Tcf1*-/- double knock-out mice [71], and Wnt signalling regulates the expression of the Notch ligand *Dll1* in the mouse PSM [72,73]. However, recent data suggest that the idea of a linear hierarchy of these pathways may be somewhat simplistic. Mouse embryos that develop in the absence of Notch activity show severely down-regulated expression of the Wnt target *mAxin2* in the PSM [33]. Similarly, *mAxin2* expression was shown to be modulated in the *Dll1* mutant mouse [53] and the Wnt-related cyclic gene *mNkd1* does not cycle in the *Hes7*-/- mouse [54]. It is likely, then, that the Notch and Wnt pathways regulate each other in a bidirectional manner in the PSM (Figure 2B). A recent study using pharmacological inhibition of either the Wnt or Notch pathway in both chick and mouse affirmed that this is indeed the case, since this treatment down-regulates PSM expression of various target genes from the reciprocal pathway [59].

The means by which Notch and Wnt interact in the PSM is currently poorly understood. Moreover, the interactions between these pathways at the level of either target gene expression or regulation of oscillation pace are not necessarily mediated by the same downstream components, further complicating matters. The fact that Wnt signalling regulates PSM expression of the Notch ligand Delta-like-1 could establish one level of cross talk. Nrarp (Notch-regulated ankyrin repeat protein) [74–76] is also a candidate to mediate this interaction. *Nrarp,* a Notch-regulated cyclic gene with dynamic expression along the PSM of fish, chick and mice embryos [77], encodes a protein that destabilizes the Notch intracellular domain (NICD) formed by cleavage of Notch upon Delta binding, but stabilizes LEF1, a key mediator of Wnt signalling, thereby participating in the regulation of both Notch and Wnt activities [75,78] (Figure 2A). If, however, *Nrarp* does mediate an interaction between Notch and Wnt in the PSM, other factors are likely to also play a role, since loss of Nrarp function does not alter the expression of cyclic genes or somite formation [77].

### Notch and Fgf

The fact that Notch and Fgf-regulated cyclic gene oscillations occur synchronously in the mouse PSM suggests that they may co-regulate each other, and/or act in synergy. Consistent with this idea, in the absence of Notch signalling, PSM expression of Fgf target genes is severely downregulated and no longer dynamic [33] (Figure 2B). Surprisingly however, dynamic *mSpry2* expression is unaffected in *RBPj*-/- embryos [56]. Note however, that, as described above, the cyclical expression of Fgf targets in these mutant mice may be a result of residual RBPj-independent Notch activity present in the PSM of these mice. This may also account for the different patterns of expression of both *mHes7* and *mSnail1* in these mutant embryos [33].

Reciprocally, mouse mutants lacking Fgf signalling display a loss of dynamic *mLfng* expression [79] (Figure 2B). Moreover, Fgf regulates *Hes7* initiation in the mouse caudal PSM [58] and *her1* and *her13.2* expression in the fish PSM [47,80], indicating that Fgf, at least in part, regulates Notch target gene expression in the PSM (Figure 2). However, the situation is not so simple, since, in the short term, oscillations of Notch-regulated cyclic genes in the chick and mouse PSM do not require Fgf signalling, and continue in its absence [59,81,82]. Further investigation will be required to clarify these observations.

### Fgf and Wnt

Expression of both *Fgf8* ligand and a variety of FGF target genes requires Wnt signalling in the PSM of both chick and mouse [53,59,60] (Figure 2B). In a reciprocal manner, the expression of the Wnt-regulated cyclic gene *mAxin2* is lost in PSM-conditional Fgfr1-/- mouse embryos or in mouse embryos treated with pharmacological inhibitors of Fgf signalling [79], indicating that the Fgf and Wnt pathways also co-regulate each other in the PSM (Figure 2B). At first glance, since the Fgf-regulated cyclic genes oscillate in synchrony with Notch-regulated genes, but asynchronously with Wnt-regulated genes, one would imagine that interactions between the Fgf and Wnt pathways or the Notch and Wnt pathways might act in a mutually inhibitory manner. However, these pathways all appear to positively regulate each other. This opens the possibility that the asynchronous oscillations are due to the timing of production, turnover and negative feedback regulation of each of the pathways following activation. Further investigation will be required to clarify if this is the case.

## Future challenges and directions

The past decade has seen remarkable progress in our understanding of vertebrate segmentation. There has also been a considerable shift in our perception of the evolutionary conservation of developmental mechanisms. However, our knowledge of the molecular mechanisms underlying the process is still very basic and limited. In the context of vertebrate segmentation, it would appear that a high degree of conservation exists, particularly in a maintained role for Notch signalling in the process (see Box 1), although this precise role may have diverged among amniotes and anamniotes. One of the challenges to be faced and resolved will be to understand the real relevance of the three signalling activities, Notch, Fgf and Wnt, in the core mechanism of the oscillator in the PSM of different vertebrate species. Another aspect that still requires a great deal of understanding is the mechanism controlling the regulation of the clock periodicity. What is the mechanism by which Wnt regulates clock pace, and which other pathway(s) also plays a role in this process? The roles of FGF8 and Wnt3a in regulating both oscillatory cyclic gene expression and in regulating the wavefront highlight a paradox, since the former relies on the oscillatory expression of target genes, while the latter relies on the non dynamic graded expression of the ligands within the same tissue. It remains to be demonstrated how these disparate functions of Wnt and FGF are both regulated and transduced by the PSM cells (Figure 1C).

In summary, although a great deal of information has become available as to the cross-talk of these three pathways during somitogenesis, it is still limited. A more complete understanding of the interaction between the Fgf, Wnt and Notch pathways, and the mechanisms in place to control segmentation clock oscillations will provide crucial information as to how the vertebrate segmented body plan is produced (see also Box 2 for a list of outstanding questions).

## Box 1 Notch and segmentation throughout the animal kingdom

In addition to the vertebrate species described in the body of the text, the Notch pathway also shows periodic signalling activity in *Xenopus* [27,28], snake [26] and lizard [26]. Remarkably, analyses of segmentation in several invertebrate species has also revealed striped expression of some Notch target genes, indicative of cyclic Notch activity in arthropods such as the spider [83], centipede [84] and cockroach [85], suggesting that Notch signalling was key to the ancestral mechanism for segmentation (reviewed in [86]). Segmentation in these species occurs *via* a progressive sequential program similar to that observed in vertebrates, and Notch inhibition in the spider or the cockroach, disrupts segmentation [83,85]. However, in certain other arthropods, such as in *Tribolium* [87] and the fruit fly [88], some Notch components are expressed in a striped segmental pattern, but are not required for segment formation. However, these insects have derived modes of development that do not reflect the ancestral insect condition. Hence, in the ancestral mode of segmentation, arthropods developed segments sequentially from a posterior growth zone, as occurs in all modern day vertebrates, and it is possible that Notch played a role in this ancestral mechanism [86].

Anamniotes are a group of vertebrates that lack the amnion during fetal development. These animals lay their eggs in water exclusively. The anamniotes comprises the Fishes and the Amphibians, the “lower vertebrates”.

Amniotes are a group of tetrapod vertebrates that have a terrestrially adapted egg. They include mammals (synapsids), and sauropsids (reptiles and birds).

## Box 2 Outstanding questions

• How does the Wnt pacemaker work?
• What other pathways modulate pace?
• What is the relevance of the individual pathways (Notch, Fgf and Wnt), in the mechanism of the oscillator in the PSM of different vertebrate species?
• Wnt regulates clock gene expression (at least in mouse), oscillation pace and the determination front; Notch regulates clock gene expression, AP compartmentalisation of prospective somites and boundary formation: How can the multiple disparate functions of these pathways in one tissue be explained [89]?
• A more complete understanding of the interaction between the Fgf, Wnt and Notch pathways is needed to understand how somitogenesis is coordinated.
